# An Investigation of the Metal Powder Ultrasound Atomisation Process of 316L Stainless Steel

**DOI:** 10.3390/ma17225642

**Published:** 2024-11-19

**Authors:** Błażej Bałasz, Łukasz Żurawski, Dorota Laskowska, Nataliya Muts, Andriana Ivanushko

**Affiliations:** 1 Faculty of Mechanical Engineering and Energy, Koszalin University of Technology, Śniadeckich 2, 75-453 Koszalin, Poland; blazej.balasz@tu.koszalin.pl (B.B.); lukasz.zurawski@tu.koszalin.pl (Ł.Ż.); 2 Department of Inorganic Chemistry, Ivan Franko National University of Lviv, Kyryla i Mefodiya Str. 6, 79005 Lviv, Ukraine; nataliya.muts@lnu.edu.ua (N.M.); andriana.ivanushko@lnu.edu.ua (A.I.)

**Keywords:** additive manufacturing, ultrasound atomisation, metal powders, stainless steel 316L

## Abstract

This paper presents the results of a study on the atomisation process of 316L material. The primary objective of the study was to obtain the highest quality, quantity and yield of 316L metal powder for the established atomisation parameters (torch current and wire feed speed) at the assumed process time. Using experiment planning according to the Taguchi method, 16 pairs of controllable variable parameters were developed. It was observed that the low current high-wire speed configuration resulted in the formation of a considerable amount of molten metal on the sonotrode platform. This phenomenon prevented the ultrasound system from working properly, so some of the processes were interrupted. The results obtained from the tests showed that torch current and wire feed speed are parameters that have a significant impact on the efficiency of the ultrasonic atomisation process. The highest efficiency was achieved by the process with a torch current of 100 A and a wire feed speed of 9 mm/s.

## 1. Introduction

Additive manufacturing (AM) involves fabricating elements by applying materials layer-by-layer according to a computer (CAD) model. Its greatest advantage lies in its ability to precisely produce elements with complex geometry in one step, which eliminates or reduces the need to connect multiple components in complex design solutions. AM also provides design freedom and flexibility, as design changes do not require significant process modifications [1,2,3]. In this way, AM enables cost reduction and time consumption for unit production or the prototyping process. Additionally, AM allows for reducing the consumption of materials by reducing waste and the possibility of its recycling, but as research shows, the number of cycles and recycling method influences the quality of manufactured components [4,5].

Additive manufacturing technologies differ mainly in the type and form of the construction material and the layer deposition technology. In binder jetting [6,7] and powder bed fusion (PBF) technologies [8,9], including selective laser melting (SLM), selective laser sintering (SLS), or electron beam melting (EBM), construction material metals and their alloys in the form of powders are used. Powders must meet several requirements regarding particle morphology, particle size, and powder size distribution (PSD) [10]. The qualities of the additive manufactured parts are significantly influenced by the characteristics of the feedstock materials [11,12,13].

Metal powder atomisation is a crucial process in the production of powders for additive manufacturing technologies [14,15,16]. There are four main technologies for producing powders for AM applications, which are as follows:

- Rotary atomisation (RA), in which molten metal is poured onto a rotating disc. Fine droplets of molten metal are ejected from the disc, solidified and collected as powders [14];
- Plasma rotating electrode process (PREP), in which droplets of molten metal are ejected from the surface of the rotating rod by centrifugal force, solidified and collected as powders [17,18];
- Water atomisation (WA), in which molten metal is sprayed and solidified by a high-pressure water jet [19];
- Gas atomisation (GA), in which molten metal is sprayed and solidified by a high-pressure flow of argon or nitrogen [19,20].

The properties of the powder depend on the manufacturing process. The average particle size of powders used in SLM technology ranges from 10 µm to 60 µm. Powders with spherical particle morphology and a wide PSD are preferred due to their flowability [21]. The flowability of fine powders results in reduced internal stresses, increased dimensional and shape accuracy, and reduced porosity and surface roughness of the manufactured object [2,22]. Powders produced by PREP technology are characterised by the best sphericity and narrow particle size distribution. However, high production costs and low process efficiency contribute to the high purchase price of the powder [23]. GA powders also exhibit spherical particle morphology, but the production cost is lower compared to PREP powders. Therefore, gas atomisation is the leading technology for the production of metal powders for AM [20].

Numerous studies have focused on the effectiveness of the GA process in producing high-quality powders. Due to the tendency to retain high-pressure gas within the molten metal, there is a high risk of gas pores or bubbles forming within the metal particles. A large number of particles with gas trapped within them increases the risk of porosity and structural defects within components produced using PBF technologies [24]. Another problem is the formation of surface satellites on the particles. Satellites are formed when solidified metal particles bounce off the bottom of the chamber and collide with the falling molten particles. Satellites reduce the flowability of the powder, which can lead to disturbances in the packing of the layer [25,26]. Consequently, alternative methods have begun to be sought. In light of the latest research, ultrasonic atomisation (UA) presents a promising alternative [27]. The UA method involves melting raw material using an electric arc. The melted material settles on the so-called sonotrode, which is connected to an ultrasound generator. Under the influence of ultrasound, the molten material is ejected from the surface of the sonotrode and sprayed by a high-pressure flow of argon. The solidified material particles are transported to the cyclotron and separated into finished atomized powder and waste [28,29].

The primary objective of the study was to obtain the highest quality, quantity and yield of 316L metal powder for the established atomisation parameters at the assumed process time. By using different characterisation methods, the efficiency of the performed processes and the suitability of the produced 316L steel powders for further applications, including PBF technologies, were assessed. On this basis, an attempt was made to estimate what variable parameters of current and wire feed speed combination allows for the acquisition of acceptable efficiency and a good quality of powder after atomisation.

## 2. Materials and Methods

### 2.1. Raw Material Characteristic

The stainless steel 316L wire with a diameter of 1.2 mm was subjected to the process of ultrasonic atomisation. The wire was produced according to PN-EN 10204:2006 [30], PN-EN 13479:2007 [31], EN ISO 9001:2015 [32] and EN ISO 14001:2015 [33] standards. Table 1 presents the chemical composition of the material, according to manufacturer certification.

**Figure 1:**
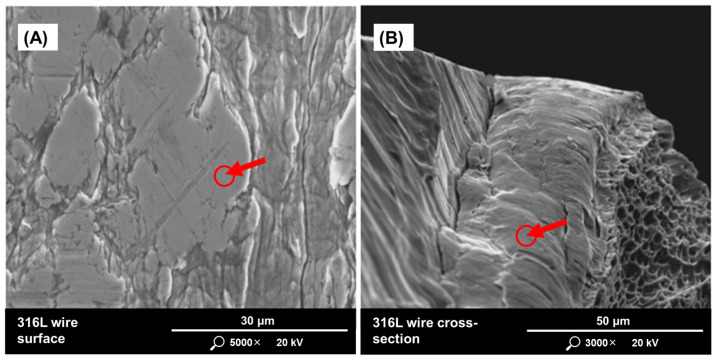
SEM images of the area of raw material for chemical composition tests where: (**A**) wire surface, (**B**) wire cross-section. The red arrows indicate the measurement location.

To verify the characteristics given by the manufacturer, the 316L stainless steel wire was examined using a Quanta 200 FEG MKII scanning electron microscope with EDS detector (FEI, Hillsboro, OR, USA). Tests were carried out on the surface (Figure 1A) and on the cross-section of the wire (Figure 1B). The results of the EDS analysis are shown in Table 2. The presented weight percentages of the detected alloying elements on the surface and cross-section of the wire differ slightly from the values given in the manufacturer’s certificate.

### 2.2. Ulrasound Atomisation

The ultrasonic atomisation process was carried out using an ATO Lab atomiser (3D Lab, Warsaw, Poland). The experimental plan was created using the Taguchi method. The variables in the experimental plan were torch current (in the range from 70 A to 150 A) and wire feed speed (in the range from 35% to 60%). Since the wire feed speed is changed as a percentage value for better analysis of raw material consumption rate, conversions to mm/s were made. It was found that 35% corresponds to 6.83 mm/s, 40% to 9.47 mm/s, 50% to 18.82 mm/s, and 60% to 28.77 mm/s. The process was carried out at a constant argon flow (60%) and a constant ultrasonic wave amplitude (80%). The ultrasound frequency ranged from 35.0 kHz to 35.3 kHz. The time of each process was 60 min. The full experimental plan is presented in Table 3.

The experimental plan assumed carrying out 16 ultrasonic atomisation processes. However, during the work, in the processes marked with symbols S5, S9, S10, S13, and S14 high instability was observed. It was most likely related to an improperly selected combination of parameters, such as too high wire feed speed or too low current. Therefore, these atomisation processes were discontinued, and the produced material was not subjected to further tests.

### 2.3. Segregation of Atomized Powder Particles

After production, the atomized material was sieved using the LPzE-3e laboratory shaker (Multiserw-Morek, Marcyporęba, Poland). For the segregation of particles with different sizes, sieves with mesh diameters of 100 μm, 80 μm, and 50 μm were used. The sieves were placed in layers, starting with the largest mesh diameter. During the single sieving cycle, each powder was divided into portions weighing approximately 50 g. The sieving time for one portion was 10 min, with the vibration amplitude set at 35% for the first 3 min, 40% for the next 3 min, and 45% for the last 4 min. After sieving each portion, the sieves were cleaned using a vacuum cleaner and compressed air. The cycle ended after the entire volume of a given powder had been sieved. For each powder, 15 sieving cycles were performed.

**Figure 2:**
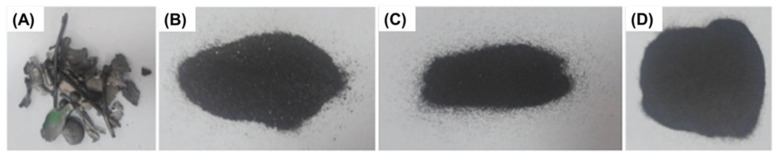
Fraction images of selected ultrasonically atomized 316L stainless steel powder: (**A**) waste, (**B**) fraction 1 (particle size ≥ 0.08 mm), (**C**) fraction 2 (particle size from 0.08 mm to 0.05 mm), (**D**) fraction 3 (particle size ≤ 0.05 mm).

In this way, each atomized powder was divided into 4 fractions (Figure 2), which were classified as follows:

- Waste (pieces of wire that have not been atomized or powder with particles size larger than 100 μm),
- Fraction 1, with particle size in the range from 80 μm to 100 μm,
- Fraction 2, with particle size in the range from 50 μm to 80 μm,
- Fraction 3, with particle size smaller than 50 μm.

Considering the requirement that powders used in powder bed fusion technologies should meet fractions 2 and 3, the atomized powders were qualified for further analysis. To clearly distinguish samples qualified for further analyses, a name coding system was introduced, which is as follows:

1. Strategy symbol;
2. Electric current intensity value;
3. Wire feed speed value;
4. Fraction symbol (F2 or F3).

After all sieving cycles, the individual fractions were weighed using a balance PS 750.R2 (RADWAG, Radom, Poland) to determine their percentage of the total weight of the powder produced in the process.

### 2.4. Atomized Powder Characteristics

A scanning electron microscope PHENOM PRO X (Thermo Fisher, Waltham, MA, USA) with an EDS detector was used to analyse the particle morphology and chemical composition of the powders.

The particle size distribution was analysed using a laser particle-size analyser ANALYSETTE 22 MicroTec Plus (Fritsch GmbH, Amberg, Germany), operating with wet dispersion technology. Particle size was also analysed from SEM images using the Phenom ParticleMetric application (Thermo Fisher, Waltham, MA, USA).

## 3. Results

### 3.1. Powder Morphology

**Figure 3:**
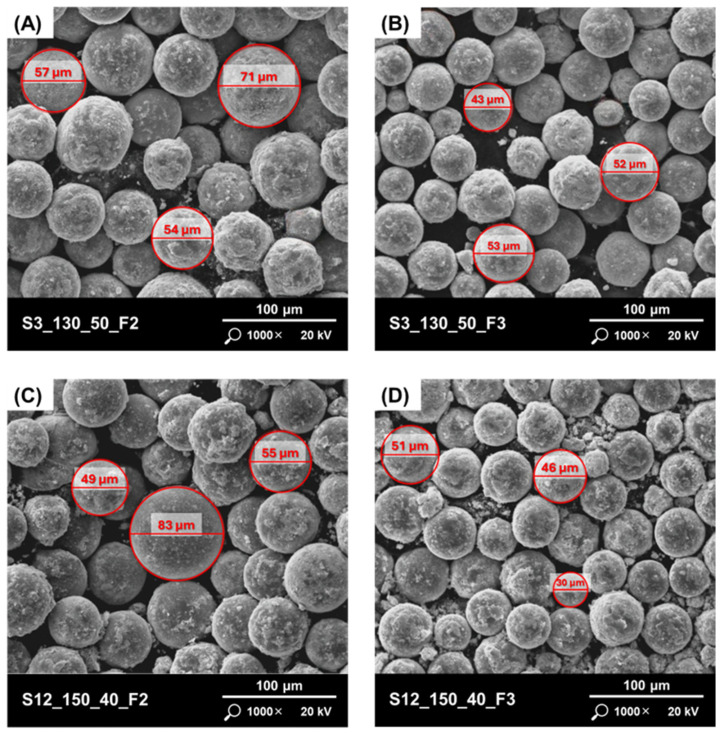
Powders with the highest irregularities in powder morphology: (**A**) S3_130_50_F2; (**B**) S3_130_50_F3; (**C**) S12_150_40_F2; (**D**) S3_150_40_F3.

Scanning electron microscopy was used to characterise the morphology and local particle features of the powders. It was found that most of the ultrasonic atomisation process strategies produced powders with spherical particles. The highest irregularities were observed for powders produced by strategies S3 (Figure 3A,B) and S12 (Figure 3C,D). The reason for this may be the high value of current and the relatively low wire feed speed.

**Figure 4:**
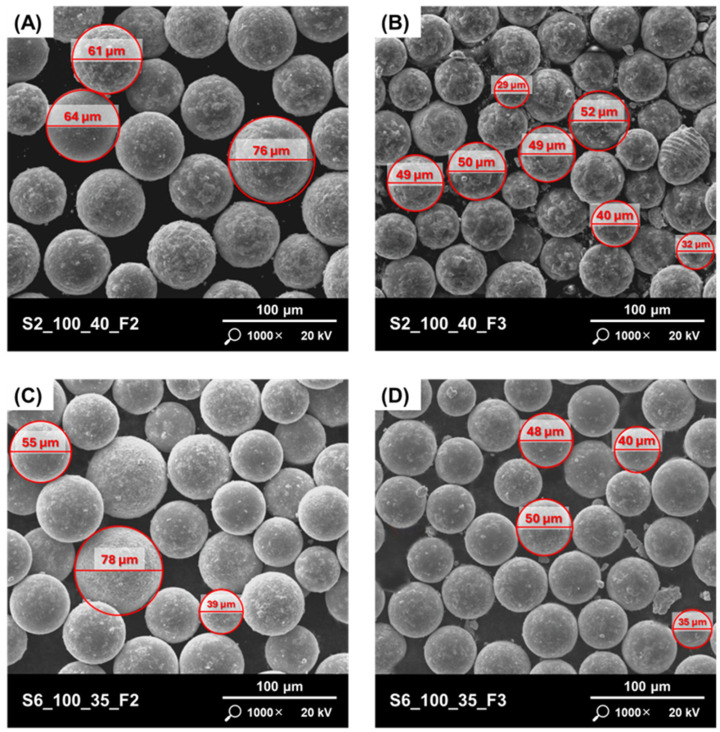
Powders with different quality of the particles surfaces: (**A**) S2_100_40_F2; (**B**) S2_100_40_F3; (**C**) S6_100_35_F2; (**D**) S6_100_35_F3.

The surface of the particles should also be noted. Various types of agglomerations are visible on the surface of the particles of the studied powders (Figure 4A,B), which may mean that during the processes, as a result of collisions, the molten material adhered to the partially or completely solidified material. No surface satellites were observed. High surface roughness can lead to a reduction in the powder flow rate, which can have a negative impact on the quality of layer packing during PBF processes. This will result in a higher number of defects (pores) inside the produced parts. The powder produced according to the S6 strategy had the lowest surface roughness and the highest particle sphericity (Figure 4C,D).

### 3.2. Particle Size Distribution

**Figure 5:**
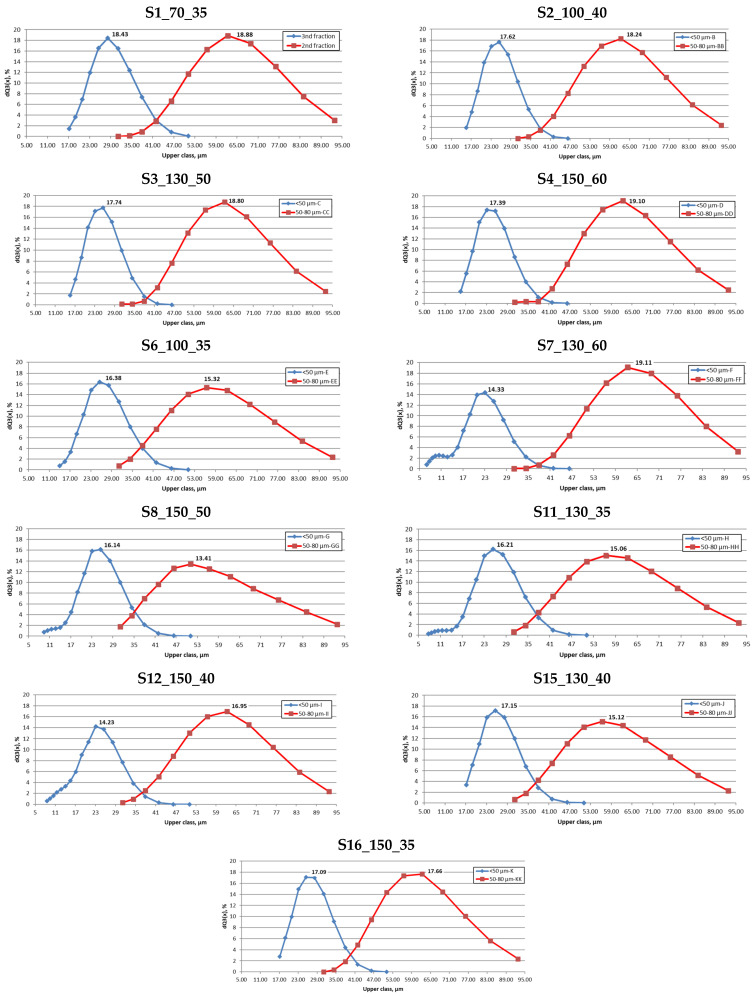
Particle size distribution of tested powders by fractions 2 and 3.

Figure 5 shows the results of the particle size distribution analysis conducted for the 2nd and 3rd fractions of the obtained powders. The tests showed that the fractions tested had a common part, that is, the particles of the same size. The results of the control measurements carried out after 5 sieving cycles showed that the common part remained at 15% dQ3(x), respectively. After all 15 sieving cycles were completed, the value dropped to between 1% and 5% of dQ3(x). Based on this, it was concluded that the adopted fraction separation method, although time-consuming, allows for the effective separation of particles. It can be assumed that performing further sieving cycles would allow full separation from fraction 2 of particles ≤50 mm in size.

It should be noted that according to the graphs shown in Figure 5, fraction 3 included particles larger than 50 mm. Wet dispersion technology by laser beam diffraction was used to measure PSD. In this technology, the powder particles perform a spinning motion, which can promote their clumping and agglomerate formation. This results in an overestimation of size due to the effect of irregular agglomerate morphology on the laser beam’s diffraction angles. In addition, although an automatic cleaning process of the apparatus is carried out after each measurement, larger particles may be deposited at the bottom of the dispersion module or in the tube system. Powder accumulated in this way can affect the result of the next measurement [34].

**Figure 6:**
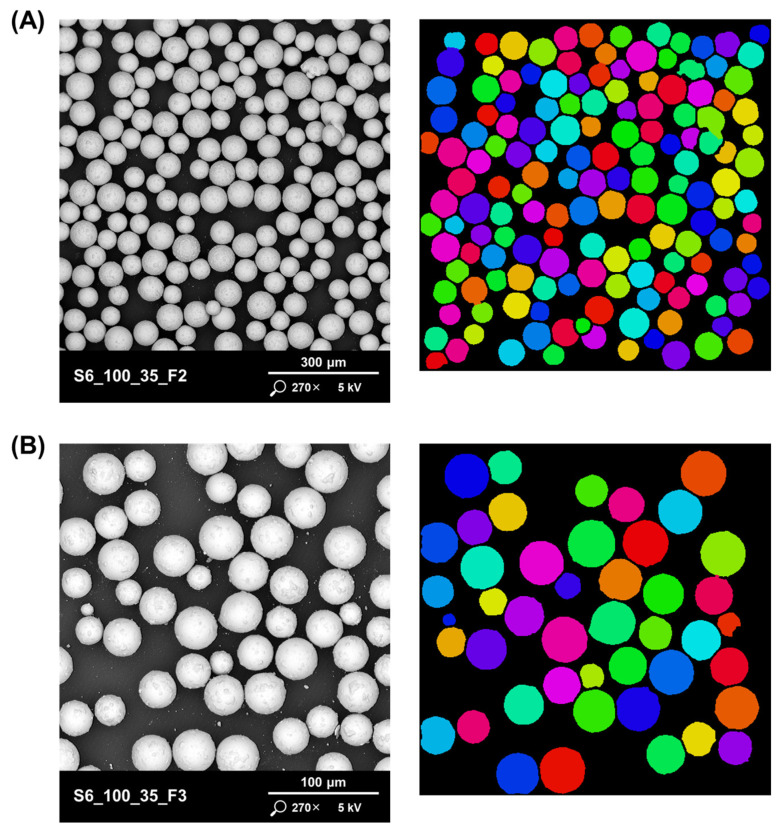
SEM image and map for statistical particle size estimation for: (**A**) S6_100_35_F2 and (**B**) S6_100_35_F3. Particles within the same size range are marked with the same colour.

The particle size distribution of the powders tested was also analysed from SEM images using the Phenom ParticleMetric application (Figure 6). The Phenom ParticleMetric application identifies individual powder particles based on the differences in grayscale and contrast between the imaged particle and the background. For particles isolated in this way, physical properties are determined, and particles of similar size are assigned the same colour to improve identification.

The analyses showed that fraction 2 of all the powders tested contained particle sizes below 50 μm. Similarly, for fraction 3 of the powders tested, particles with a size above 50 μm were mainly found. Table 4 shows the results of the analysis for the selected powder labelled as S6_100_35 (the best from the point of view of the imaging studies carried out earlier).

### 3.3. Chemical Composition of Atomized Powder

The chemical composition of the atomized powder was determined using a scanning electron microscope with an EDS detector. The obtained results are presented in Table 5.

The powders produced differed in the content of the individual alloying elements. These ranged from 59.43% to 63.85% for Fe, 17.99% to 19.53% for Cr, 9.89% to 11.22% for Ni, 3.00% to 3.53% for Mo and 1.08% to 3.88% for Mn. O was also detected on the surface of the particles, ranging from 3.35% to 4.14%. On this basis, it can be concluded that during the period between manufacturing and testing, its partial oxidation occurred.

The Fe and Cr contents were comparable for all powders and were within the range allowed by PN-EN 10088-1:2014 [35]. For the properties of 316L stainless steel, the content of Cr, which is the main alloying element responsible for corrosion resistance, is particularly important. The Ni content for all powders was below the desired values. However, it should be noted that this fact also applies to the raw material (wire). The contents of Fe, Cr and Ni were slightly lower than those of the raw material (wire), indicating the partial evaporation of these elements during the atomisation process.

Some of the powders tested showed a slightly increased content of alloying elements such as Mn and Mo. This was the case for powders from the S1, S3, S4, S7, S12 and S15 series. Powders from the S2, S6, S8, S11 and S16 series only showed an increased Mo content. For powders of these series, agglomerates were found on the surface of the particles. The powder from the series S6 showed the closest content of alloying elements to the allowable limit.

The increased content of alloying elements in the powder can be an advantage from the point of view of PBF processes. The high temperature of the melt pool results in the evaporation of elements. Considering the melting and boiling temperatures of the elements making up the chemical composition of 316L stainless steel, evaporation of Mn will occur first. As research shows, Mn is the element responsible for stabilising the austenitic phase in 316L steel [36]. The increased content of alloying elements in the powder will preserve the appropriate content of alloying elements in the solidified component, contributing to the preservation of properties similar to material produced by traditional techniques.

### 3.4. Analysis of Atomisation Efficiency

Table 6 presents an analysis of the performance of the atomisation processes carried out, taking into account the length (l_wire_) and mass (m_wire_) of the raw material used (wire), the total mass of powder produced (m_powder_) and the proportion of each fraction in the total mass of powder produced. The duration of each process was 60 min.

The highest efficiency, understood as the ratio of the total mass of powder produced (m_powder_) to the mass of raw material used (m_wire_), was in the S6_100_35 process, at 80.6%.

A laboratory shaker was used to separate the different powder fractions, together with a set of sieves with the appropriate mesh size. After all 16 sieving cycles had been completed, it was found that fraction 2 (containing particles between 80 μm and 50 μm in size) accounted for 48% to 56% of the total weight of the material produced. Fraction 3 (containing particles < 50 μm in size) accounted for 30% to 42% of the total mass of material produced. The atomisation process should also be assessed by the percentage of the F1 and waste fractions. Minimising the proportion of these fractions, especially waste, is important, as they will not be used in further technological processes. This criterion is best met by powders produced according to strategies S6_100_35 and S3_130_50. However, the latter had the lowest yield, at 32.5%.

## 4. Conclusions

The aim of the study was to obtain the highest quality, quantity and yield of 316L metal powder for the established atomisation parameters at the assumed process time. Based on the performed characteristics, it was found that:

1. Torch current and wire feed speed are parameters are parameters that have a significant impact on the efficiency of the ultrasonic atomisation process. The highest efficiency was achieved by the process labelled as S6, with torch current 100 A and the wire feed speed 9 mm/s.
2. For some combinations of variable parameters, the atomisation process was highly unstable. This was manifested by the formation of a mass of liquid metal on the sonotrode surface, which interfered with the correct operation of the ultrasound generating system.
3. All performed ultrasonic atomisation processes allowed obtaining powders with similar and almost spherical grain morphology.
4. The applied fraction separation method allowed for the separation of powder fractions with grain sizes and PSD dedicated to PBF processes.
5. After repeated sieving of the powder for all performed atomisation processes, approximately 53% of the fraction with grain size in the range from 50 to 80 ang, approximately 36% of the fraction with grain size smaller than 50 μm were obtained.
6. The chemical compositions of the obtained powders differed slightly from the values described by the standards. The most satisfactory chemical composition was characterised by the powder marked as S6.

Further experimental studies will involve the use of 316L steel powders obtained in LPBF technology. Their aim will be to investigate the influence of the type of material (from ultrasound atomisation versus gas atomisation) used on selected properties of the manufactured elements.

## Figures and Tables

**Table 1:** Chemical composition of the raw material stainless steel 316L wire.

Element	Fe	Cr	Ni	Mo	Mn	Si	C
**Weight percent [%]**	64	19	11.5	2.75	1.75	0.70	<0.025

**Table 2:** Chemical composition of raw material-stainless steel 316L wire.

Material	Element Weight Percent [%]					
	Fe	Cr	Ni	Mo	Mn	Si
316L wire surface	65.11	18.88	12.05	3.19	2.16	0.77
316L wire cross-section	62.70	18.34	12.17	3.51	2.37	0.90

**Table 3:** Experiment plan parameters of ultrasound atomisation process.

Strategy Symbol	Atomized Powder Sample Symbol	Parameters		
		Torch Current [A]	Wire Feed Speed	
			[%]	[mm/s]
1	S1_70_35	70	35	6.83
2	S2_100_40	100	40	9.47
3	S3_130_50	130	50	18.82
4	S4_150_60	150	60	28.77
5 *	S5_70_40	70	40	9.47
6	S6_100_35	100	35	6.83
7	S7_130_60	130	60	28.77
8	S8_150_50	150	50	18.82
9 *	S9_70_50	70	50	18.82
10 *	S10_100_60	100	60	28.77
11	S11_130_35	130	35	6.83
12	S12_150_40	150	40	9.47
13 *	S13_70_60	70	60	28.77
14 *	S14_100_50	100	50	18.82
15	S15_130_40	130	40	9.47
16	S16_150_35	150	35	6.83

* Excluded due to process instability.

**Table 4:** Statistical particle size estimation obtained base of SEM image.

Property	Material	
	S6_100_35_F2	S6_100_35_F3
Circle equivalent diameter [μm]	62.50	41.13
Major axis [μm]	64.10	42.57
Minor axis [μm]	61.00	39.80
Circumference [μm]	198.00	132.33
Convex hull [μm]	197.00	131.50
Circumscribed circle diameter [μm]	67.20	44.90

**Table 5:** Chemical composition (weight percentage [%]) of atomized powder.

Atomized Powder Sample Symbol	Fe	Cr	Ni	Mo	Mn	O
S1_70_35	61.27	18.19	11.22	3.24	2.11	3.98
S2_100_40	61.28	18.86	10.75	3.53	1.34	3.68
S3_130_50	60.95	18.32	10.60	3.36	2.82	3.93
S4_150_60	59.43	19.53	10.01	3.00	3.88	4.14
S6_100_35	63.26	17.99	10.77	3.41	1.08	3.49
S7_130_60	60.67	19.43	9.55	3.10	3.42	3.83
S8_150_50	61.80	19.12	10.27	3.26	1.95	3.83
S11_130_35	63.70	18.20	10.31	3.42	1.08	3.36
S12_150_40	61.51	18.56	10.32	3.30	2.48	3.83
S15_130_40	62.12	19.07	9.89	3.08	2.27	3.58
S16_150_35	63.85	18.13	10.03	3.25	1.33	3.35

**Table 6:** Analysis of atomisation processes efficiency.

Strategy Symbol	l_wire_ [cm]	m_wire_ [g]	m_powder_ [g]	Efficiency [%]	Fractions Contribution							
					F3		F2		F1		Waste	
					[g]	[%]	[g]	[%]	[g]	[%]	[g]	[%]
S1_70_35	2460	222	137	61.7	50.1	36.5	65.7	48.0	3.7	2.7	5.6	4.1
S2_100_40	3420	309	204	66.0	73.5	36.0	105.4	51.7	4.7	2.3	15.9	7.8
S3_130_50	6780	613	199	32.5	83.8	42.1	103.1	51.8	7.8	3.9	3.2	1.6
S4_150_60	10,380	938	562	59.9	204.6	36.4	310.3	55.2	18.7	3.3	21.4	3.8
S6_100_35	2460	222	179	80.6	71.6	40.0	94.4	52.7	2.9	1.6	2.1	1.2
S7_130_60	10,380	939	690	73.5	258.2	37.4	354.9	51.4	29.0	4.2	38.0	5.5
S8_150_50	6780	613	453	73.9	137.1	30.3	244.4	54.0	28.9	6.4	23.5	5.2
S11_130_35	2460	222	136	61.3	49.4	36.3	75.6	55.6	2.4	1.8	6.1	4.5
S12_150_40	3420	309	212	68.6	69.5	32.8	108.5	51.2	7.4	3.5	17.8	8.4
S15_130_40	3420	309	227	73.5	76.5	33.7	124.8	55.0	4.3	1.9	14.1	6.2
S16_150_35	2460	222	135	60.8	45.4	33.6	72.4	53.7	1.5	1.1	6.1	4.5

Where: F3-particles size smaller than 50 μm; F2-particles size in range from 50 μm to 80 μm; F1-particles size in range from 80 μm to 100 μm; waste-particles size larger than 100 μm.

## Data Availability

The raw data supporting the conclusions of this article will be made available by the authors on request.
